# Effect of Glucocorticoid Treatment on Computed Tomography Angiography Detected Large-Vessel Inflammation in Giant-Cell Arteritis. A Prospective, Longitudinal Study

**DOI:** 10.1097/MD.0000000000000486

**Published:** 2015-02-06

**Authors:** Sergio Prieto-González, Ana García-Martínez, Itziar Tavera-Bahillo, José Hernández-Rodríguez, José Gutiérrez-Chacoff, Marco A. Alba, Giuseppe Murgia, Georgina Espígol-Frigolé, Marcelo Sánchez, Pedro Arguis, Maria C. Cid

**Affiliations:** From the Vasculitis Research Unit, Departments of Systemic Autoimmune Diseases (SP-G, IT-B, JH-R, MAA, GM, GE-F, MCC); Emergency Medicine (AG-M); and Radiology (JG-C, MS, PA), Hospital Clínic, University of Barcelona, Institut d’Investigacions Biomèdiques August Pi i Sunyer (IDIBAPS).

## Abstract

Computed tomography angiography (CTA) detects signs of large-vessel vasculitis (LVV) in about 67.5% of patients with giant-cell arteritis (GCA) at the time of diagnosis and early aortic dilatation in 15%. The outcome of CTA-findings of LVV upon glucocorticoid treatment has not been prospectively evaluated.

The aim of our study was to prospectively assess glucocorticoid-induced changes in CTA findings of LVV in patients with GCA.

Forty biopsy-proven GCA patients evaluated by CTA at diagnosis were prospectively followed and scheduled a new CTA approximately after 1 year of treatment. Vessel wall thickening, diameter, and contrast enhancement of the aorta and its tributaries were evaluated. Results were compared to those obtained at the time of diagnosis.

CTA was repeated to 35 patients after a median follow-up of 13.5 months (IQ25–75% 12.4–15.8). Arterial wall thickening was still present in 17 patients (68% of the patients who initially had LVV). The number of affected segments and wall thickness at various aortic segments significantly decreased and no patients developed new lesions, new aortic dilation or increase in previous dilation. Contrast enhancement disappeared in 15 (93.75%) of 16 patients in whom this finding could be assessed.

Signs of LVV improve with treatment. While contrast enhancement resolves in the majority of patients, vessel wall thickening persists in two thirds. However, the number of affected aortic segments as well as aortic wall thickness significantly decreases. Longer follow-up is necessary to determine the clinical significance of persisting wall thickening and its relationship with relapses or subsequent development of aortic dilatation or large-vessel stenoses.

## INTRODUCTION

Giant-cell arteritis (GCA) is a systemic granulomatous vasculitis affecting large- and medium-sized vessels.^1–3^ Although GCA was initially considered as a vasculitis mainly involving the carotid and vertebral artery branches, prospective imaging studies have recently demonstrated that widespread large-vessel involvement is common.^4–13^

The prevalence of large-vessel inflammation in newly diagnosed patients has been evaluated in a few prospective studies only, using ^18^fluoro-deoxyglucose-positron emission tomography (FDG-PET), computed tomography angiography (CTA), or color duplex ultrasonography Frequency ranges from 29% to 74% of patients, depending on the vascular territory explored and the technique employed.^4,6,8–12^

Inflammatory involvement of the aorta has been evaluated in three sizable prospective imaging studies using FDG-PET or computed tomography, with or without angiography,^4,6,7^ and ranges from 45% to 65% of patients with GCA at the time of diagnosis. Moreover, retrospective studies have shown that, during follow-up, dilation of the aorta, particularly in the thoracic segment, is significantly more frequent in GCA patients than in the general population.^14–17^ Furthermore, a cross-sectional systematic screening of 54 patients with GCA demonstrated that aortic dilatation occurred in 12 (22.5%) after a median follow-up of 5.4 years.^18^ An extended follow-up of this cohort disclosed that 33.3% of patients develop aortic dilatation after a median of 10.3 years.^19^ To date, the specific mechanisms leading to aortic dilatation in GCA are unclear. It is conceivable and assumed that aortic dilatation is a consequence of previous inflammation but a direct link between initial inflammation and subsequent dilatation has not been established.

It has been hypothesized that aortic dilatation results from persistent remaining subclinical aortic inflammation.^20,21^ However, surgical or necropsy specimens from aortic aneurysms, usually obtained months or years after diagnosis, not always disclose persistent inflammatory infiltrates or these are residual.^18–20^ Consequently, early damage of the elastic fibers and muscular layer by inflammation, inefficient vascular repair or remodeling after injury, vascular ageing, and/or hemodynamic factors may also contribute to aneurysm development, regardless of whether or not active inflammation has resolved.^3,4,16,22,23^

In this study, we prospectively evaluated the outcome of CTA signs of large-vessel inflammation and remodeling in GCA patients after approximately 1 year of glucocorticoid treatment. Specific aims of our survey were (1) to investigate the persistence or resolution of CTA findings suggesting vascular inflammation (wall thickening and contrast enhancement) compared to the initial evaluation,^4^ (2) to assess the development of signs of structural damage or remodeling (dilation and/or stenosis), and (3) to identify clinical or blood test data associated with resolution or persistence of CTA signs of vascular inflammation.

## PATIENTS AND METHODS

### Patients

The initial cohort included 40 patients who had been evaluated by CTA at the time of GCA diagnosis in order to detect large-vessel involvement, as part of a prospective study.^4^ These patients were treated and followed by the investigators according to a defined protocol and tapering schedule^18,24–26^ and were appointed a new CTA examination between 12–18 months after the initiation of glucocorticoid treatment. Two patients were lost to follow-up (1 patient due to severe dementia and 1 patient because of unknown reasons) and 3 declined a new CTA. Follow-up CTA was completed in the remaining 35 patients.

Clinical and exploratory findings were prospectively recorded at the time of diagnosis, and at 1, 3, 6, 9, 12 month-follow-up and at the time of CTA imaging. The recorded items were: cranial symptoms (headache, scalp tenderness, and jaw claudication), polymyalgia rheumatica, systemic symptoms (weight loss, and fever), disease-related cranial ischemic complications, and presence of extremity claudication. Acute phase reactants including C-reactive protein (CRP), erythrocyte sedimentation rate (ESR), and hemoglobin were also determined at every visit. A combination of clinical and blood test abnormalities was used to evaluate the intensity of the systemic inflammatory response (SIR) as previously reported.^18^ These included fever >37 °C, weight loss >3 kg, hemoglobin (Hb) <11 g/L, and erythrocyte sedimentation rate (ESR) ≥85 mm/h. Patients with 3 or 4 of these items were considered to have a strong SIR, whereas patients with ≤2 were considered to have a weak SIR.

### CTA Protocol

CTA was performed using a multi-slice CT scanner (Somatom Definition Flash, Siemens Medical Solutions, Erlangen, Germany) with the following scanning conditions: collimation 0.6 mm, 120 kV, mAs determined by automatic modulation dose, and reconstruction slice thickness of 5.0 and 1 mm. One hundred milliliter of non-ionic contrast agent (370 mg I/ml) was injected through the ante-cubital vein using a power injector at the rate of 4 ml/s. Early arterial and late venous phases were acquired. We used the bolus-tracking method at the descending aorta level and set 100  Hounsfield units (HU) as the triggering threshold for the arterial phase. The venous phase scan was taken 60 seconds after the arterial phase was completed. Significant enhancement of the aortic wall was considered when an increase of 20 HU or more existed between the arterial and venous phases.

**FIGURE 1:**
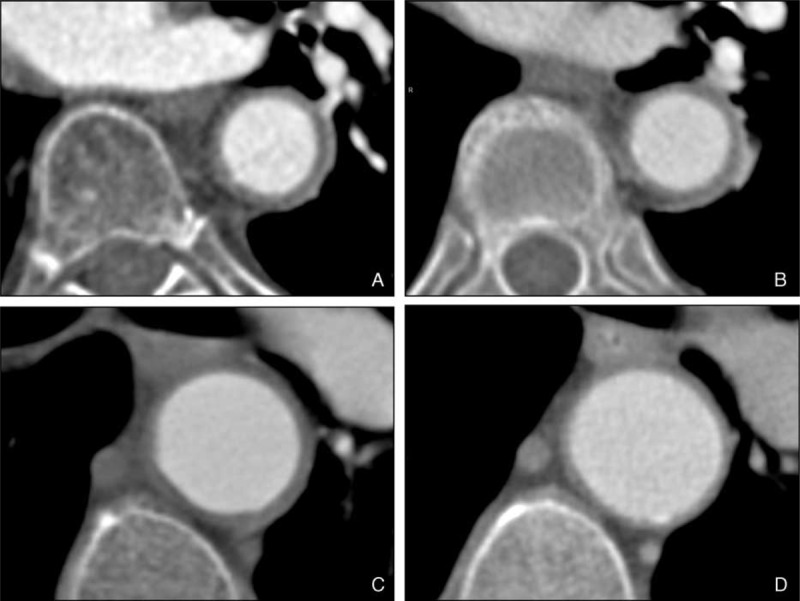
CTA of a newly diagnosed patient with GCA showing significant aortic wall thickening (panel A) with contrast enhancement in the late venous phase, sparing the intimal layer (panel B). Persistent aortic wall thickening after 1-year of treatment, devoid of contrast enhancement in the late venous phase (panels C and D).

Vascular dilatation or stenosis, as well as vessel wall thickening, were assessed at 4 aortic segments (ascending thoracic aorta, aortic arch, descending thoracic aorta and abdominal aorta) in all patients. Moreover, in 16 patients, the presence of significant wall thickening and appropriate and timely imaging acquisition permitted evaluation of contrast enhancement of the aortic wall at both assessments. As in the first evaluation,^4^ aortitis was defined as circumferential aortic wall thicknening ≥2 mm observed in zones without adjacent atheroma. In addition, in the present study, contrast enhancement was evaluated, when feasible, in both CTA assessments, in order to distinguish between inflammatory activity (presence of contrast enhancement) and vascular remodeling (absence) (Figure 1).^4,27–29^ Aortic dilatation was defined by a diameter >4 cm in the ascending aorta, ≥4 cm in the rest of the thoracic aorta, and ≥3 cm in the abdominal aorta. Loss of the physiologically progressive diameter reduction of the aorta was also considered dilatation.^18,30,31^

The aortic tributaries, including brachiocephalic trunk, carotid, subclavian, axillary, splanchnic (celiac and mesenteric), renal, iliac, and femoral arteries, were also evaluated. Radiological findings of large-vessel inflammation were circumferential wall thickness >1 mm and contrast enhancement of the artery wall. Signs of abnormal large-vessel remodeling were arterial dilatation, and the presence of focal stenoses not related to atheroma.^4^

CTA evaluation was performed by the same radiologists (PA and JGC), blinded to the clinical and laboratory data.

### Statistical Analysis

Mann–Whitney *U* test and Student's *t* test, for paired or independent data when applicable, were used for quantitative data. Fisher's exact test was applied to contingency tables for qualitative data. Bonferroni correction was applied to multiple comparisons. Calculations were performed with the IBM SPSS Statistics (Version 20.0, Armonk, NY).

## RESULTS

### Clinical and Laboratory Findings

Of the 35 patients finally included, 25 were women and 10 men with a mean age of 79 ± 6 years (mean ± SD). Median follow-up time between both radiologic evaluations was 13.5 months (IQ25–75% 12.4–15.8).

Although two patients relapsed during the study period, at the time of the second CTA, all patients were in clinical remission and receiving glucocorticoid treatment, with a median prednisone dose of 5 mg/day (ranging from 10 mg/day, due to a recent relapse, to 2.5 mg/day). No specific symptoms or signs of large-vessel stenosis (bruits, asymmetry in arm blood pressure or extremity claudication) were referred or detected. The main clinical, laboratory and treatment data of the study cohort are summarized in Table 1.

**TABLE 1:**
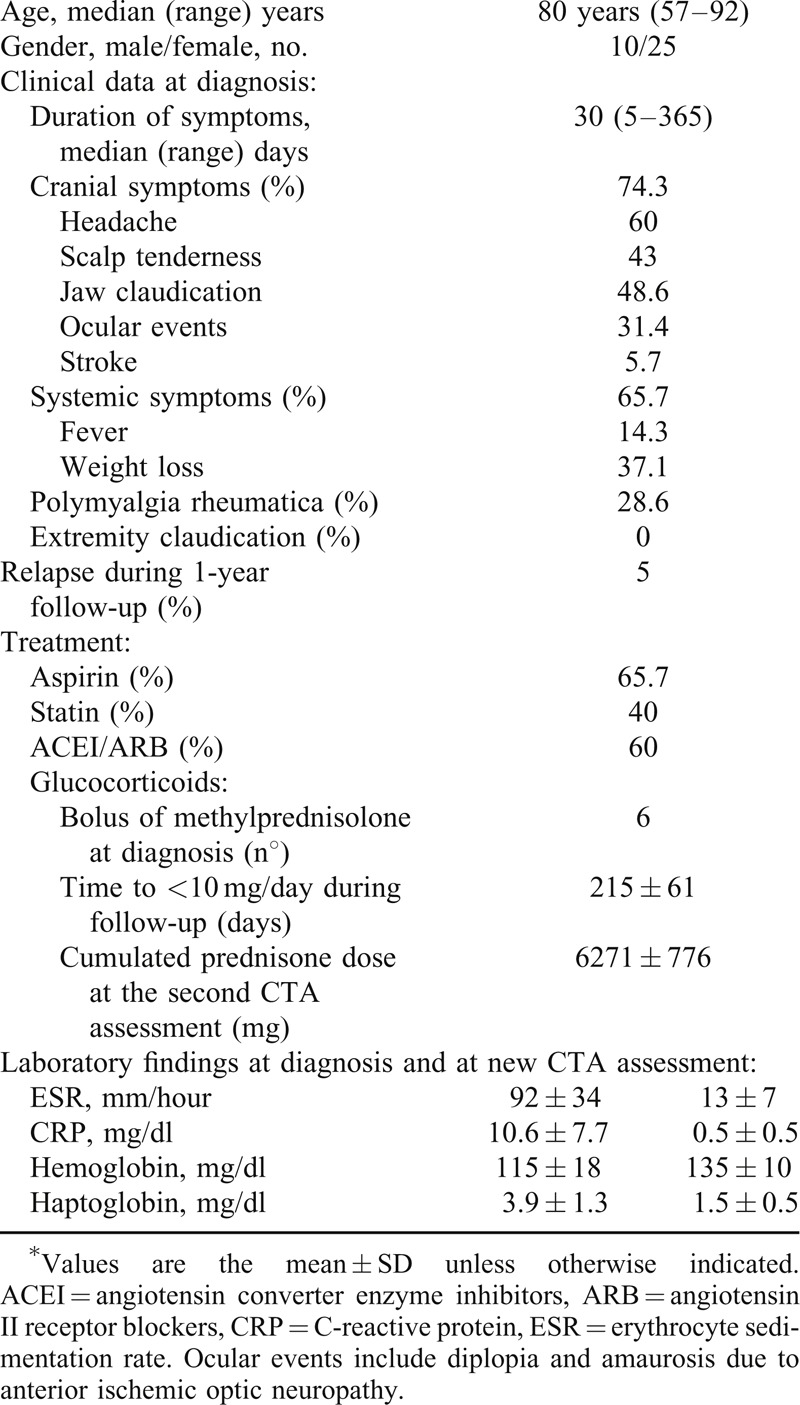
Clinical and Biochemical Findings at Diagnosis and Treatment Data of the Investigated GCA Cohort of 35 Patients

### Prevalence and Topography of Large-Vessel Involvement After 1-Year Follow-Up

**FIGURE 2:**
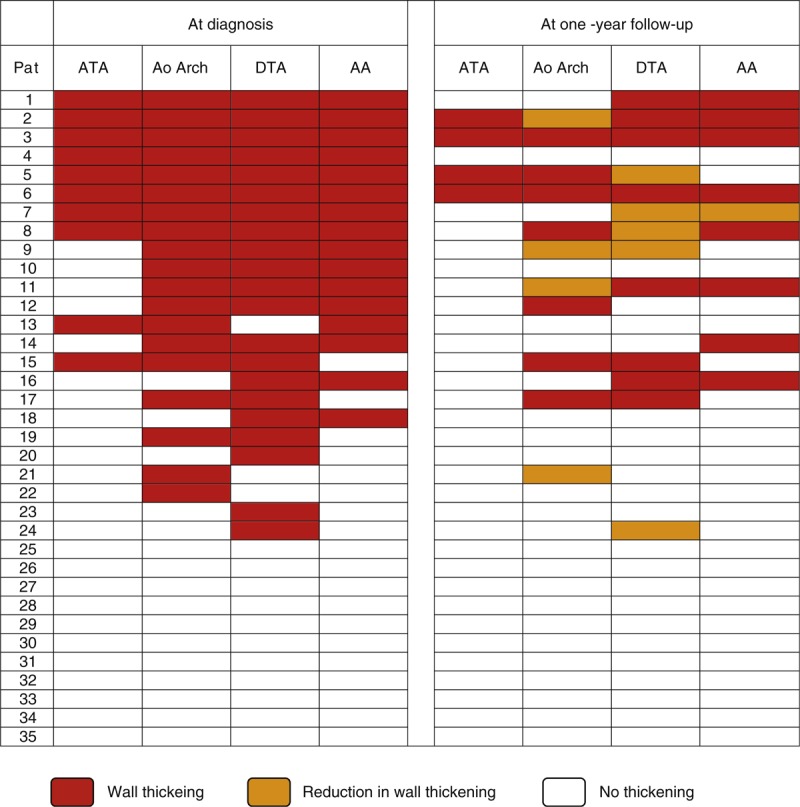
Distribution of the aortic wall thickening in the GCA cohort at both CTA assessments. ATA = ascending thoracic aorta; DTA = descending thoracic aorta; AA = abdominal aorta; Ao = aortic.

Nineteen out of the 35 patients (54.3%) had abnormal CTA findings. Radiological signs suggesting LVV (wall thickening) were still seen in 17 patients (48.5%) and aortic dilation in 5 (14.3%). As in the first evaluation, the descending thoracic aorta and the aortic arch were the most affected segments: aortic thickening was observed in 13 (37.1%) of patients at the descending thoracic aorta and in 11 (31.4%) of patients at the aortic arch, followed by the abdominal aorta and the ascending aorta, that were involved in 9 (25.7%) and in 4 (11.4%) of patients, respectively. Furthermore, CTA-defined aortic branch inflammation was still detected in 12 (34.2%) patients. Detailed frequencies of radiological LVV at the different vascular segments evaluated and its distribution in the aorta at both evaluation times are summarized in Table 2 and Figure 2, respectively. Topography of the aortic segments with persistence, reduction or resolved radiological inflammation is depicted in Figure 2.

**TABLE 2:**
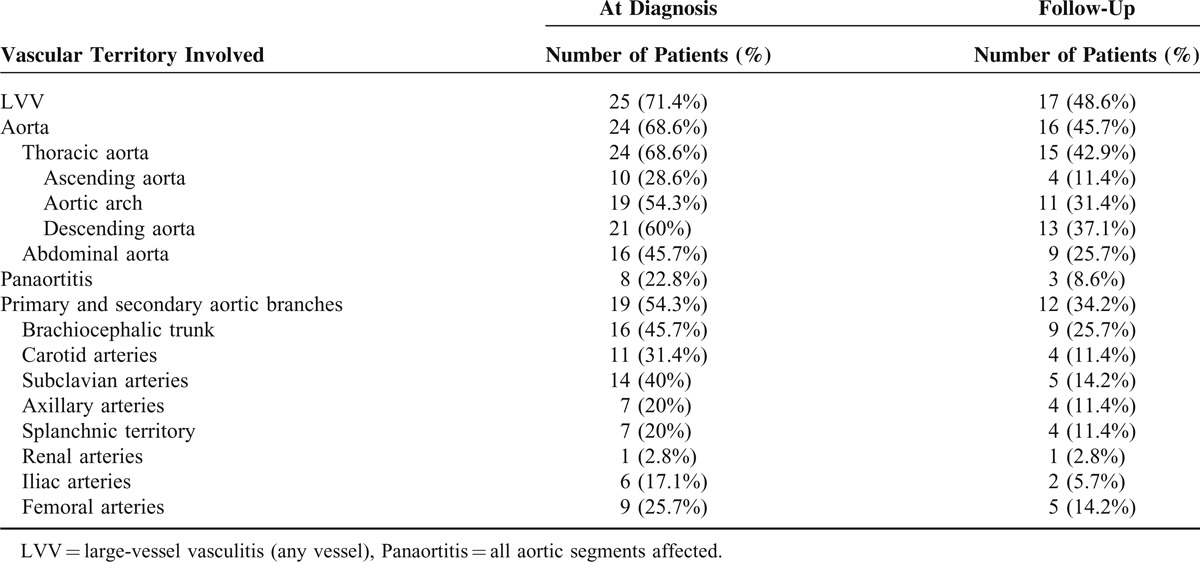
Topography of Inflammatory Large-Vessel Involvement in the Study Cohort at the Time of Diagnosis and at the Follow-Up Evaluation

**FIGURE 3:**
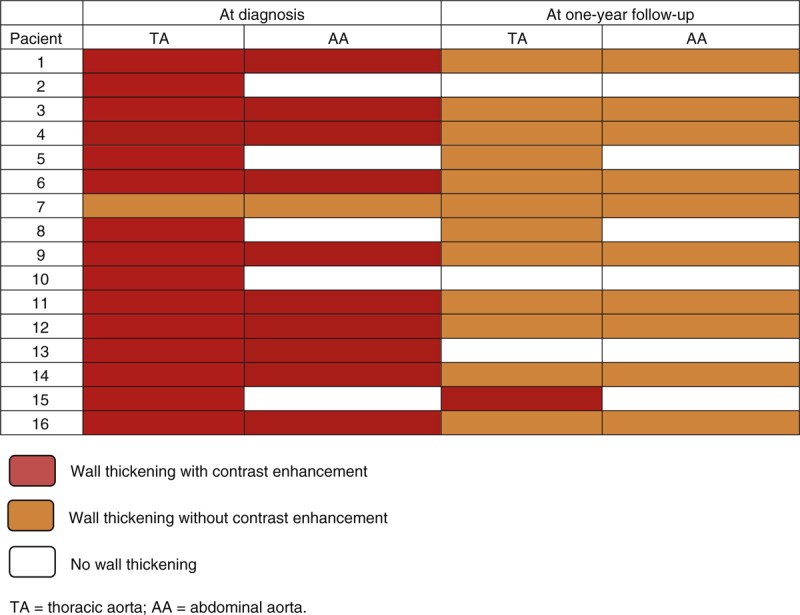
Distribution of the contrast enhancement in the followed GCA cohort at both radiologic assessments. TA = thoracic aorta; AA = abdominal aorta.

When comparing both evaluations, initial and present, vessel wall thickening was still present in 17 (68%) of the 25 patients who initially had this finding. A significant reduction in the mean aortic wall thickness was detected in all aortic segments (Table 3). Moreover, the number of vascular segments affected substantially decreased and none of the 35 patients developed new inflammatory lesions in previously unaffected areas (Figure 2). Post-hoc evaluation of the 16 patients in whom contrast enhancement assessment was feasible, disclosed that all patients but 1 (93.75%) had contrast enhancement of the aortic wall as a sign of active vasculitis^27–29^ in the first CTA evaluation performed at the time of GCA diagnosis. Conversely, at the second assessment, contrast enhancement was evident in the aortic wall in only one (6.25%) of the 16 evaluable patients (see Figure 3).

**TABLE 3:**
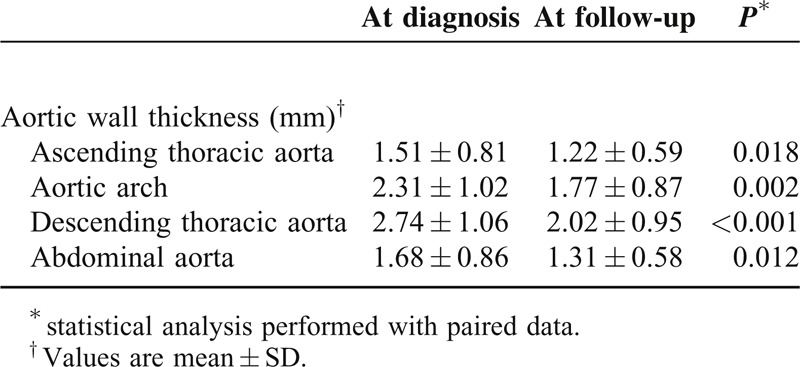
Aortic Wall Thickness at Diagnosis and at the follow-Up Assessment

There were no significant differences in aortic diameters when comparing both evaluations. No new aortic dilatation was detected and, moreover, dilatation detected at the ascending thoracic aorta in 5 patients at the time of GCA diagnosis, remained stable. At the first assessment, only one patient showed a vascular stenosis, concomitant with wall thickening, located in the left renal artery. At the second assessment, these remained stable and another patient developed a new vascular stenosis in a segment located at the inferior mesenteric artery with no previously apparent CTA inflammatory signs.

### Clinical, Laboratory and Treatment Features Associated With Persistence or Resolution of CTA Signs of Large-Vessel Inflammation

Twenty-five out of the 35 patients who completed both evaluations had signs of LVV at the time of diagnosis. Of those, 17 had persisting signs of LVV at follow-up whereas vessel wall thickening had resolved in the remaining 8. The time lapse between both CTA assessments was similar in patients with or without persisting LVV at follow-up. No significant differences in age, gender or clinical symptoms were found between both groups (Table 4). Only 2 patients suffered from relapse, 1 in each group.

**TABLE 4:**
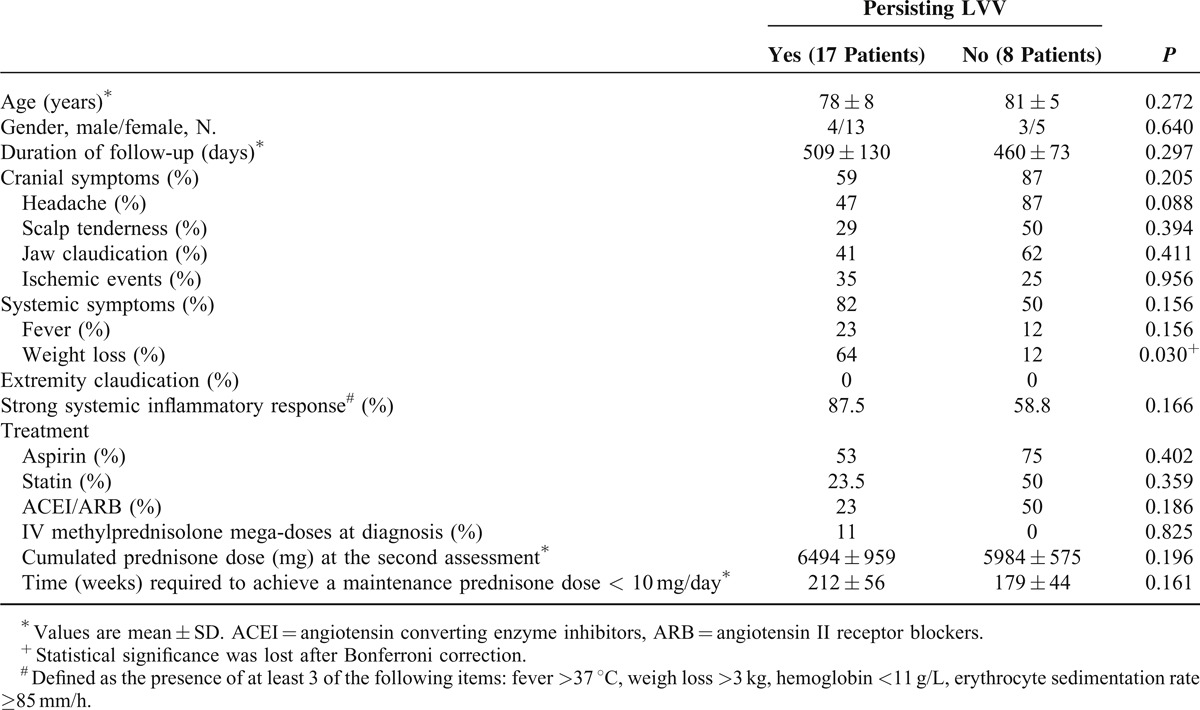
Clinical Features at Baseline and Treatments Received by Patients With Persisting or Resolved Large-Vessel Thickening at the Second CTA Evaluation

**FIGURE 4:**
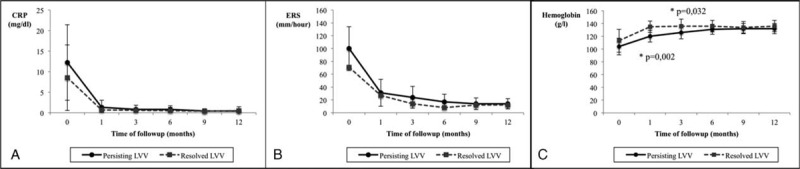
Acute phase reactants at scheduled time points during 1-year follow-up of patients with or without persisting LVV at the second assessment.

Acute phase reactants at the specified time-points during follow-up in patients with or without persisting signs of LVV at the second CTA are shown in Figure 4. Only hemoglobin was significantly lower at the first follow-up visits in patients with persisting vessel wall thickening. When comparing acute phase reactants in patients in whom wall thickening persisted unchanged versus those who experienced improvement, only ESR was significantly higher in patients with unmodified wall thickening. No relationship was found between the presence of a strong or weak SIR at diagnosis and the outcome of the imaging abnormalities during follow-up (Table 4).

**FIGURE 5:**
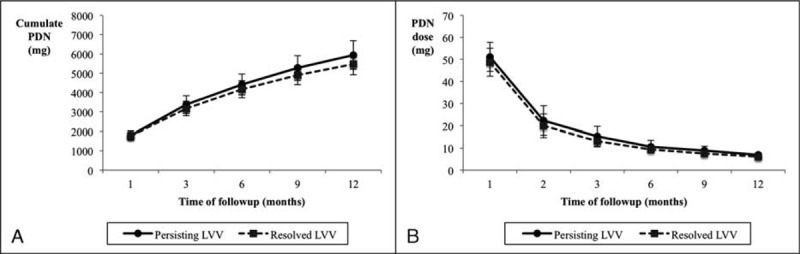
Cumulated prednisone dose (A) and daily prednisone dose (B) at scheduled time points during follow-up in patients with persisting or resolved LVV.

Data regarding patients’ therapy are summarized in Table 4 and Figure 5. There were no significant differences in the percentage of patients who had received IV methyl-prednisolone megadoses at diagnosis because of cranial ischemic manifestations, or other medications including aspirin, statins, or angiotensin-converting enzyme inhibitors /angiotensin II receptor blockers between both groups. There were no differences either in the time required to achieve a maintenance prednisone dose below 10 mg/day, in the cumulated prednisone dose at the second imaging between patients with persisting or resolved vessel wall thickening or in the prednisone doses at the different follow-up visits.

## DISCUSSION

Recent prospective studies have revealed an elevated prevalence of inflammation of the aorta and its primary and secondary branches in GCA.^4–13^ However, the clinical impact of large-vessel inflammation detected at diagnosis on patient's outcome is unclear. Population-based studies have demonstrated an increased incidence of thoracic aortic aneurysm in GCA.^14,15,17,32^ However, a direct link between aortic inflammation and later development of aortic dilatation seems reasonable but it has not been clearly established. Male gender, smoking habit, and hypertension have been associated with higher risk of aortic aneurysm in patients with GCA in some studies.^17,18,33^ Blockmans et al demonstrated, indeed, larger aortic diameters detected by computed tomography (CT) in a heterogeneous cohort of GCA patients who had increased aortic FDG uptake in a PET performed 1–110 months before.^34^ Moreover, although there are reports of symptomatic stenoses of primary and secondary branches of the aorta leading to extremity claudication,^35,36^ mesenteric ischemia,^37,38^ myocardial infarction,^39^ or ischemic stroke,^40,41^ the percentage of patients who develop large-vessel stenosis is unknown and the relationship between stenosis and previous inflammatory involvement has not been demonstrated. It is estimated that between 5% and 15% of patients have signs or symptoms derived from large-vessel stenosis at diagnosis or during follow-up.^32,35,42,43^ However, since studies addressing this point are retrospective and not all patients were imaged, it is difficult to distinguish between signs and symptoms derived form atherosclerotic vascular disease, which is not unusual in the age group targeted by GCA, from those derived from vasculitic involvement.

To date, prospective longitudinal studies in unselected cohorts of GCA patients in order to define the outcome of large-vessel inflammation have not been reported. This is the first study prospectively evaluating the early outcome of CTA defined large-vessel vasculitis detected at the time of diagnosis in a cohort of patients with biopsy-proven GCA subjected to a standardized glucocorticoid treatment in accordance with international recommendations.^44^ We evaluated both the outcome of vessel wall thickening suggestive of vasculitis as well as changes in the vessel diameter indicating dilatation or stenosis after approximately 1 year of follow-up.

Unexpectedly, given the complete clinical response to treatment in the majority of patients, persistent vessel wall thickening was found in 48.5% of patients, two-third of the patients who had signs of LVV at the time of diagnosis. However, vessel wall thickening as well as the number of affected vessels significantly decreased and there was no new involvement of previously spared segments, indicating, to a certain extent, a substantial response of large-vessel inflammation to glucocorticoid therapy. Moreover, it has been repeatedly remarked that the capacity of imaging techniques to detect signs of large-vessel inflammation quickly decreases after gluococorticoid treatment.^45–48^ The significance of persistent vessel wall thickening during glucocorticoid treatment is unclear. It may represent persistent subclinical, pauci-symptomatic vascular inflammation, refractory to maintenance glucocorticoid therapy, which would strongly underline the need for more efficient therapies able to abrogate silent large-vessel inflammation.^49^ However, it is noteworthy that patients were in clinical remission with normal or close to normal acute phase reactants at the time of the second assessment. Moreover, no major differences in serum concentrations of acute phase reactants over time was observed between patients with resolved large-vessel involvement and those with persisting vascular thickening. Therefore, major persistence of inflammatory activity was unlikely.

Second temporal artery biopsies have been performed to some patients with GCA several months or years after the initiation of glucocorticosteroid treatment for a variety of reasons: including confirmation of a clinically based diagnosis, necropsy, or as part of an ancillary study in the context of an international multicentre clinical trial.^50–53^ In these specimens, scattered small foci of inflammatory cells persist but the more striking finding consists of prominent medial fibrosis and vascular wall remodeling. It is likely that persistent vascular wall thickening primarily represents fibrosis and vascular remodeling rather than major persistence of active inflammation. This is supported by the observation of contrast enhancement in only one out of 16 patients in whom this finding could be assessed. Concomitant PET scan may have been useful to differentiate active inflammation and scarring in patients with persisting vascular thickening as recently suggested for patients with cardiac involvement by eosinophilic granulomatosis with polyangiitis.^12,54^

No changes in aortic diameter indicative of dilatation were observed when comparing the initial and the follow-up assessments. This observation supports the concept that aortic dilatation is a delayed complication as suggested by retrospective, population-based studies^14,15,17,32^ and a cross-sectional study with its long-term follow-up.^18,19^

Regarding to vascular stenosis, only 1 patient developed reduction in the inferior mesenteric artery diameter and no patients had clinical signs or symptoms related to vascular stenosis in the second assessment indicating that the development of stenosis in the primary or secondary branches of the aorta is infrequent in the early outcome of unselected patients with GCA. Given the imaging modality used (CTA), conclusions about stenosis in more distal territories such as infrapopliteal arteries, which may be involved by GCA,^3,9,55–58^ cannot be drawn. These data underlines important differences with Takayasu disease. Based on the observation that GCA frequently targets large vessels, it has been recently proposed that GCA and Takayasu disease may be the edges of the spectrum of a single disorder and that clinico-pathological differences may be related to ageing of the immune and/or vascular system.^59^ Although in the absence of an identified etiology discussing whether or not Takayasu and GCA are different conditions or the same disease may be inconclusive, our observations support that there are important differences in vascular remodelling between both conditions. While patients with Takayasu disease frequently and typically develop vascular stenoses, sometimes in spite of immunosuppressive therapy, vascular lumen reduction is unusual in unselected patients with GCA.

Our study has the strengths of its prospective nature and careful imaging analysis of the vascular territories accessible to CTA. It has also some limitations including the relatively small size of the patient cohort and the short-term follow-up. Extended studies are necessary to delimitate the long-term outcome and clinical impact of large-vessel inflammation in GCA.
